# Rapid-Onset Myositis and Myocarditis Following Dual Immune Checkpoint Inhibitor Therapy With Nivolumab and Ipilimumab for Gastric Adenocarcinoma: A Case Report

**DOI:** 10.7759/cureus.84121

**Published:** 2025-05-14

**Authors:** Steven B Barker, Adeoluwa Adewuyi

**Affiliations:** 1 Internal Medicine, Northeast Georgia Medical Center Gainesville, Gainesville, USA

**Keywords:** adenocarcinoma-gastric type, immune checkpoint inhibitors, immune-related adverse event (irae), immune therapy-mediated myocarditis, immune therapy-mediated myositis, ipilimumab-related adverse events, nivolumab-related adverse events

## Abstract

Immune checkpoint inhibitors (ICIs) have revolutionized oncology by improving survival outcomes with fewer side effects than traditional chemotherapy. However, ICIs are associated with immune-related adverse events (irAEs) affecting various organ systems, including rare neuromuscular complications such as myositis and myocarditis. This report presents a 70-year-old male with gastric adenocarcinoma, Lynch syndrome, and other comorbidities who developed severe myositis and myocarditis, confirmed by MRI and enzyme testing, 11 days after his first cycle of nivolumab and ipilimumab. He initially showed improvement with high-dose steroids, but he declined further treatment. Due to his myositis, he suffered respiratory decline, cardiac dysfunction, and intensive care unit admission. Despite urgent plasma exchange and resumed steroid therapy, his condition did not improve, and care was transitioned to comfort measures to honor the patient’s wishes. This case underscores the challenges in managing severe irAEs, particularly in steroid-refractory cases, where advanced immunosuppressive strategies may be needed. Early recognition of symptoms and multidisciplinary coordination are crucial for mitigating complications. As ICIs become more widespread, clinicians must remain vigilant for life-threatening irAEs and balance aggressive treatment with patient autonomy.

## Introduction

In recent years, immune checkpoint inhibitors (ICIs) have rapidly changed the field of oncology, providing therapies with improved survival benefits for a wide variety of cancers in the neoadjuvant and adjuvant settings [1]. One advantage of ICIs is that many patients experience fewer treatment-related adverse effects and improved safety profiles compared to traditional chemotherapy [2,3]. However, the use of ICIs has been associated with its own spectrum of immune-related adverse events (irAEs) affecting multiple organ systems [4]. Neuromuscular adverse effects are a less common subset of irAEs that have previously been reported using ICIs [5-8]. Among neuromuscular irAEs, one study showed that myositis is the most common with concurrent myocarditis occurring in 32% of cases [8]. The median time of onset of symptoms in this study was 19 weeks [8]. In one case report consisting of two cases of fulminant myocarditis, symptom onset in both instances was 12 and 15 days after starting nivolumab and ipilimumab combination therapy [9]. Rapid symptom onset, like in our case, is rare and only described in a few case reports [9]. In general, irAEs are managed with high-dose steroids. 

Here, we present a case of a patient with gastric adenocarcinoma who developed ICI-induced myositis and myocarditis within 11 days of his first treatment with programmed cell death protein 1 (PD-1) and cytotoxic T-lymphocyte-associated protein 4 (CTLA-4) dual immunotherapy. 

## Case presentation

A 70-year-old Caucasian male presents to an outpatient oncology follow-up with new-onset neck weakness and diffuse muscle pain eleven days after his first dose of dual ICI therapy with nivolumab and ipilimumab. Before this office visit, the patient was diagnosed with Siewert type 3 gastroesophageal junction adenocarcinoma and began treatment with eight cycles of neoadjuvant chemotherapy using the FLOT regimen (5-fluorouracil, leucovorin, oxaliplatin, and docetaxel). During his initial treatment with the FLOT regimen, molecular testing revealed high microsatellite instability (MSI-H) and a mutation in MutS Homolog 2 (MSH2), consistent with Lynch syndrome. After completing eight cycles of FLOT, surveillance imaging showed minimal tumor response. Given the poor radiographic response and the tumor’s MSI-H status, the patient was transitioned to neoadjuvant dual ICI therapy with nivolumab and ipilimumab, in accordance with National Comprehensive Cancer Network guidelines for resectable mismatch repair-deficient gastric cancer [10], with plans for tumor resection at a later date.

**Figure 1:**
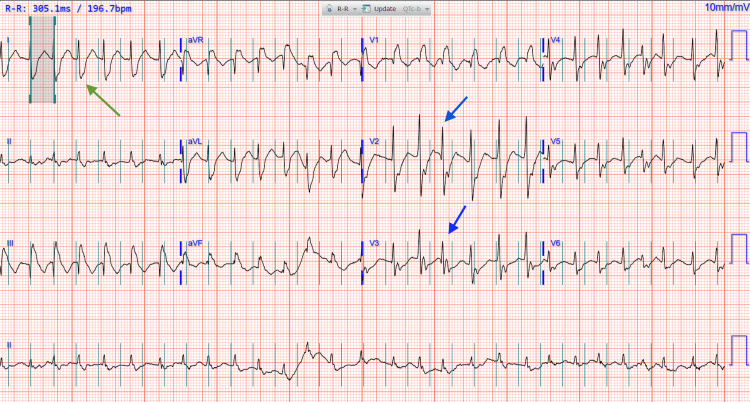
ECG on admission showing atrial fibrillation with RVR, RBBB, and nonspecific T-wave changes. Gray lines indicate an irregularly irregular rhythm with absent P waves, consistent with atrial fibrillation. The blue arrow highlights a wide QRS complex with an RSR' pattern in leads V2 to V3, indicative of RBBB. The green arrow indicates biphasic QRS complexes with a prominent S wave in lead I, also consistent with RBBB. RVR, rapid ventricular response; RBBB, right bundle branch block; ECG, electrocardiogram

On presentation, physical examination revealed symmetric proximal muscle weakness, ptosis, and hyporeflexia in the lower extremities. Laboratory tests showed a total creatine kinase (CK) level of 12,419 U/L, troponin I of 13,113 ng/L, and creatine kinase-myocardial band (CK-MB) of 302.4 ng/mL. An electrocardiogram (ECG) showed atrial fibrillation with rapid ventricular response (RVR), right bundle branch block (RBBB), and nonspecific T-wave abnormalities (Figure 1). A transthoracic echocardiogram on admission revealed a normal left ventricular ejection fraction (LVEF), and a review of medical records was notable for a recent cardiac stress test showing no ischemia. Labs (Table 1) demonstrated transaminitis, elevated N terminal-pro brain natriuretic peptide (NT-proBNP), and negative viral hepatitis antibodies. He was mildly anemic and had a mild leukocytosis. MRI of the brain and spine was notable for symmetric edema in the posterior paraspinal musculature of the cervical, upper thoracic, and lumbar spine, suggesting localized, selective myositis.

**Table 1:** Pertinent lab values from admission (CMP, CBC, NT-proBNP, COVID-19/influenza/RSV, TSH/T4/T3, and hepatitis panel) and during hospitalization (aldolase, MuSK antibody, and acetylcholine receptor antibody). CMP, comprehensive metabolic panel; CBC, complete blood count; NT-proBNP, N terminal-pro brain natriuretic peptide; RSV, respiratory syncytial virus; TSH, thyroid-stimulating hormone; T4, thyroxine; T3, triiodothyronine; MuSK, muscle-specific kinase; CK-MB, creatine kinase-myocardial band; CK, creatine kinase

Lab	Value	Reference Range
Basic Labs		
BUN	29.0 mg/dL	5-23 mg/dL
Creatinine	0.95 mg/dL	0.80-1.30 mg/dL
AST	858 U/L	0-48 U/L
ALT	469 U/L	13-61 U/L
Alkaline Phosphatase	106 U/L	45-136 U/L
Hemoglobin	12.2 mg/dL	14.0-18.0 mg/dL
Hematocrit	40.7%	42.0-52.0%
MCV	75.5 fL	80.0-94.0 fL
Platelets	369 K/µL	130-400 K/µL
White Blood Cells	11.9 K/µL	4.8-10.8 K/µL
TSH	1.158 µIU/mL	0.350-4.940 µIU/mL
Free T4	1.00 ng/dL	0.71-1.48 ng/dL
Free T3	1.77 pg/mL	1.45-3.48 pg/mL
COVID-19/influenza A&B/RSV	All Negative	Negative
Hepatic Labs		
Hepatitis B Surface Ag	Negative	Negative
Hepatitis A IgM	Negative	Negative
Hepatitis B Core IgM	Negative	Negative
Hepatitis C Ab	Negative	Negative
Musculoskeletal Labs		
Aldolase	215.0 U/L	1.2 -7.6 U/L
Acetylcholine Receptor Ab	0.00 nmol/L	≤0.02 nmol/L
MuSK Autoantibody	0.00 nmol/L	0.00-0.02 nmol/L
Cardiac Labs		
CK	12,419 U/L	46.00-171.00 U/L
CK-MB	302.4 ng/mL	0.0-5.0 ng/mL
High Sensitivity Troponin	13,113 ng/L	<45 ng/L
NT-proBNP	9,988 pg/mL	<125 pg/mL (Age <75)

**Figure 2:**
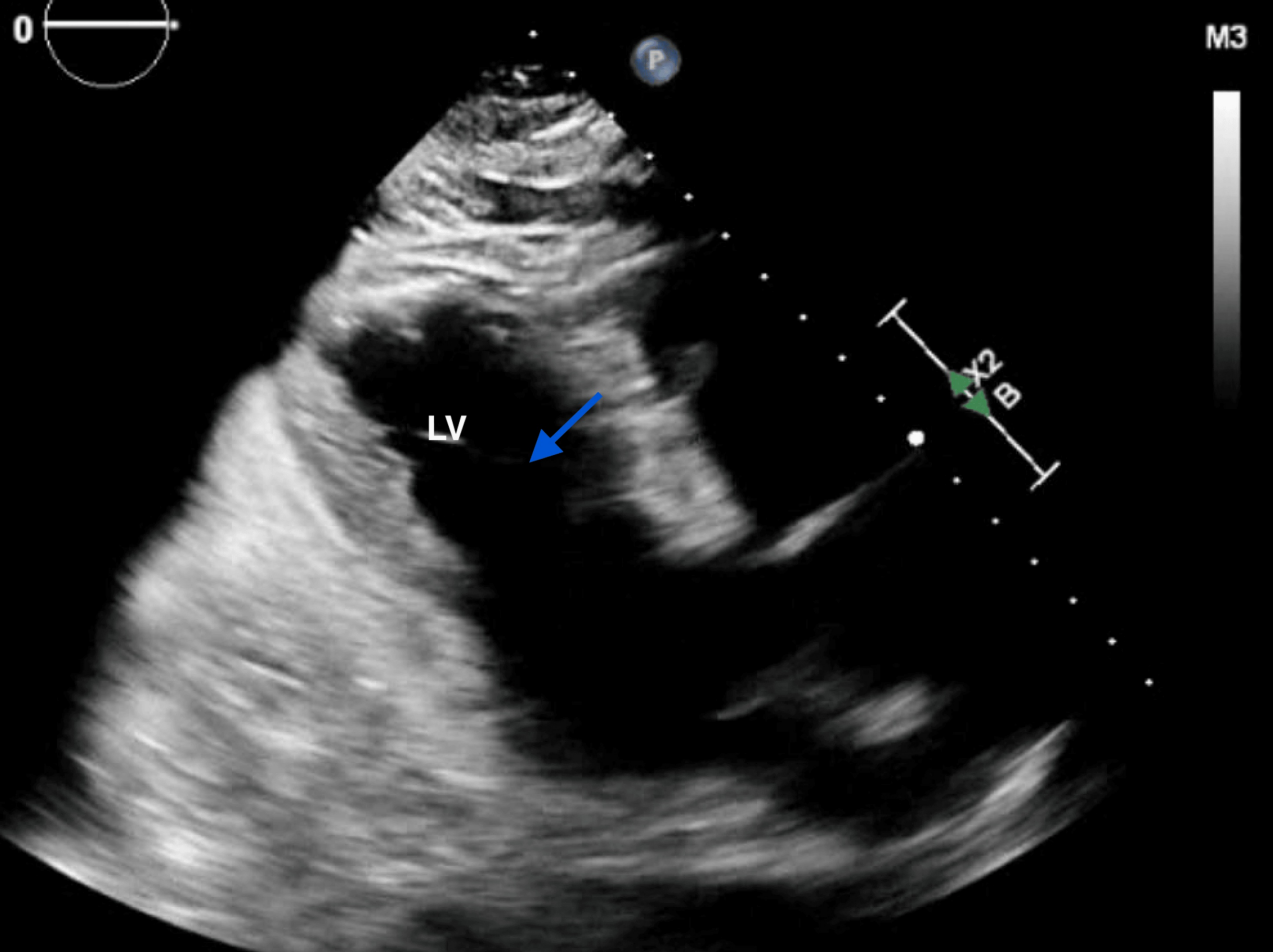
Transthoracic echocardiogram (parasternal long-axis view) at maximal left ventricular contraction, showing reduced ejection fraction and elevated end-systolic volume. The blue arrow denotes increased end-systolic volume. LV, left ventricle

The patient's symptoms initially improved after one dose of steroids, but the steroids caused insomnia. He declined further treatment with steroids due to insomnia, despite being offered medications to manage it, following informed shared decision-making regarding his prognosis without steroids or immunomodulatory therapies. On hospital day four, he decompensated with respiratory failure and shock, requiring intubation and vasopressors after a suspected aspiration event. He was transferred to the intensive care unit. Arterial blood gas showed respiratory acidosis; repeat echocardiography showed a newly reduced LVEF of 30-35% (Figure 2) compared to an LVEF of 55-60% just one month prior. Repeat labs were obtained during his intensive care unit course to re-assess his myositis and were unchanged from his initial presentation. Due to his worsening condition, surgical resection of his gastric adenocarcinoma was deferred in favor of medical stabilization and further testing to differentiate between irAE myositis and myasthenia gravis, given his presentation with ptosis. Antibody panels were obtained (Table 1) and were negative for myasthenia gravis. A diagnostic muscle biopsy was offered for a definitive diagnosis; however, he declined the biopsy. Right upper quadrant ultrasound revealed cholelithiasis and steatosis, but no obstruction was seen. A chest x-ray revealed aspiration pneumonia.

Despite the re-initiation of steroids and empiric plasma exchange [11], respiratory parameters, including negative inspiratory force (NIF), did not improve. His NIF values were 0, 0, 0, -5, -3, -1, and -1 on intensive care unit days one to seven, respectively. After two sessions of plasma exchange and a seven-day stay in the intensive care unit, the patient and his family, following a shared decision-making discussion with the care team, opted for comfort-focused care due to his overall prognosis, which remained poor given the minimal improvement in respiratory function despite high-dose steroids and plasma exchange.

## Discussion

This case of immune-related myositis and myocarditis illustrates the complexity of managing irAEs, where both clinical judgment and ethical considerations play a critical role. Diagnostic tools include cardiac biomarkers, serum aldolase, electrocardiography, echocardiography, cardiac magnetic resonance, and endomyocardial biopsy [12]. Although a biopsy was not performed, the diagnosis of myositis is supported by a combination of clinical and diagnostic findings. In the appropriate clinical context, as in this case, elevated serum aldolase, MRI evidence of muscle inflammation, characteristic symptoms of weakness, and laboratory markers of muscle breakdown can collectively establish the diagnosis when biopsy is not feasible.

Immune-related myositis is a rare adverse event, with an incidence of 0.38% [13], and is associated with ICI combination therapy, such as the combination of nivolumab and ipilimumab used in this case [14]. This condition is thought to be T-cell-mediated, as evidenced by the fact that ICI irAE myositis tests negative for autoimmune antibodies seen in other neuromuscular syndromes [15]. The spectrum of irAEs is broad and potentially life-threatening, involving multiple organ systems including the musculoskeletal, respiratory, dermatologic, hepatic, endocrine, and gastrointestinal systems [15,16]. In severe cases, myositis can extend to the cardiac and respiratory muscles [15]. While some patients may fully recover with steroid therapy and resume immunotherapy [10], others may experience fatal outcomes [16]. Managing immune-related myositis remains challenging, and further research is needed to optimize treatment strategies. 

The primary treatment of immune-related myositis and myocarditis involves discontinuing ICIs and initiating high-dose corticosteroids (1-2 mg/kg/day of prednisone or other steroid of equivalent dosing) with a slow steroid taper over four to six weeks [17,18]. However, approximately half of patients do not respond to corticosteroids alone, necessitating additional immunosuppressive therapies [17]. Case reports have shown success with combinations such as abatacept and mycophenolate mofetil for steroid-refractory cases [18]. However, these treatments have not yet shown mortality benefits. Mortality rates for ICI-induced myocarditis remain high, especially in severe cases (50%) [19].

In our case, symptom onset started rapidly compared to the previously reported 19-week median onset of symptoms in immune-related myositis and myocarditis [8]. Although the full scope of management for irAEs was offered to our patient, he declined those therapies in favor of a comfort-based approach, which was most consistent with his goals of care. Palliative care was offered. This case certainly highlights the ethical principle of patient autonomy that guides clinicians in the care of their patients daily. While healthcare providers endeavor to provide our patients with evidence-based medicine, we must acknowledge and respect the transcendent ethical principles that govern our care. Shared decision-making conversations are crucial to ensuring our patients are fully informed, understand their diagnosis and prognosis, and have the support they need in autonomous or proxy decision-making in the case of incapacitation. In this case, shared decision-making conversations resulted in a decision to focus on compassionate comfort care. 

## Conclusions

Serious irAEs, such as myositis and myocarditis, are rare and typically occur around 19 weeks after treatment initiation. Our case represents a rare instance of rapid-onset, severe immune-related myositis and myocarditis occurring just 11 days after the first dose of combination therapy. As the indications for ICIs increase, we can expect to see more irAEs. This case highlights the need for heightened awareness among oncologists, hospitalists, and other multidisciplinary team members involved in the care of those receiving ICIs. As more cases of irAEs are described, clinicians must be able to promptly recognize ICI irAEs, such as myositis and myocarditis, to optimize patient outcomes and ensure the safe continuation of immunotherapy in appropriate cases. Finally, this case highlights the importance of collaboration between the patient and the multidisciplinary care team, emphasizing the prioritization of patient autonomy and the evolving balance between medical innovation and individualized care.

## References

[1] Bagchi S, Yuan R, Engleman EG (2021). Immune checkpoint inhibitors for the treatment of cancer: clinical impact and mechanisms of response and resistance. Annu Rev Pathol.

[2] Reck M, Rodríguez-Abreu D, Robinson AG (2016). Pembrolizumab versus chemotherapy for PD-L1-positive non-small-cell lung cancer. N Engl J Med.

[3] Robert C, Long GV, Brady B (2015). Nivolumab in previously untreated melanoma without BRAF mutation. N Engl J Med.

[4] Ramos-Casals M, Brahmer JR, Callahan MK (2020). Immune-related adverse events of checkpoint inhibitors. Nat Rev Dis Primers.

[5] Solimando AG, Crudele L, Leone P (2020). Immune checkpoint inhibitor-related myositis: from biology to bedside. Int J Mol Sci.

[6] Martins F, Sofiya L, Sykiotis GP (2019). Adverse effects of immune-checkpoint inhibitors: epidemiology, management and surveillance. Nat Rev Clin Oncol.

[7] Bajwa R, Cheema A, Khan T (2019). Adverse effects of immune checkpoint inhibitors (programmed death-1 inhibitors and cytotoxic T-lymphocyte-associated protein-4 inhibitors): results of a retrospective study. J Clin Med Res.

[8] Moreira A, Loquai C, Pföhler C (2019). Myositis and neuromuscular side-effects induced by immune checkpoint inhibitors. Eur J Cancer.

[9] Johnson DB, Balko JM, Compton ML (2016). Fulminant myocarditis with combination immune checkpoint blockade. N Engl J Med.

[10] (2024). National Comprehensive Cancer Network - Clinical Practice Guidelines in Oncology: gastric cancer. https://www.nccn.org/professionals/physician_gls/pdf/gastric.pdf.

[11] Safa H, Johnson DH, Trinh VA (2019). Immune checkpoint inhibitor related myasthenia gravis: single center experience and systematic review of the literature. J Immunother Cancer.

[12] Hamada N, Maeda A, Takase-Minegishi K (2021). Incidence and distinct features of immune checkpoint inhibitor-related myositis from idiopathic inflammatory myositis: a single-center experience with systematic literature review and meta-analysis. Front Immunol.

[13] Okubo N, Kijima T, Nukui A, Kamai T (2020). Immune-related myositis resulting from combination therapy of ipilimumab and nivolumab in patient with metastatic renal cell carcinoma. BMJ Case Rep.

[14] Sutaria R, Patel P, Danve A (2019). Autoimmune myositis and myasthenia gravis resulting from a combination therapy with nivolumab and ipilimumab for metastatic melanoma. Eur J Rheumatol.

[15] Mehta A, Gupta A, Hannallah F, Koshy T, Reimold S (2016). Myocarditis as an immune-related adverse event with ipilimumab/nivolumab combination therapy for metastatic melanoma. Melanoma Res.

[16] Alnabulsi R, Hussain A, DeAngelis D (2018). Complete ophthalmoplegia in Ipilmumab and Nivolumab combination treatment for metastatic melanoma. Orbit.

[17] Jespersen MS, Fanø S, Stenør C, Møller AK (2021). A case report of immune checkpoint inhibitor-related steroid-refractory myocarditis and myasthenia gravis-like myositis treated with abatacept and mycophenolate mofetil. Eur Heart J Case Rep.

[18] Schneider BJ, Naidoo J, Santomasso BD (2021). Management of immune-related adverse events in patients treated with immune checkpoint inhibitor therapy: ASCO guideline update. J Clin Oncol.

[19] Heemelaar JC, Louisa M, Neilan TG (2024). Treatment of immune checkpoint inhibitor-associated myocarditis. J Cardiovasc Pharmacol.

